# Effects of protein-rich nutritional supplementation and bisphosphonates on body composition, handgrip strength and health-related quality of life after hip fracture: a 12-month randomized controlled study

**DOI:** 10.1186/s12877-015-0144-7

**Published:** 2015-11-17

**Authors:** Lena Flodin, Tommy Cederholm, Maria Sääf, Eva Samnegård, Wilhelmina Ekström, Amer N. Al-Ani, Margareta Hedström

**Affiliations:** Department of Geriatric Medicine, Karolinska University Hospital, Stockholm, Sweden; Department of Public Health and Caring Sciences, Clinical Nutrition and Metabolism, Uppsala University, Uppsala, Sweden; Department of Endocrinology, Metabolism and Diabetes, Karolinska University Hospital, Stockholm, Sweden; Division of Orthopedics, Department of Clinical Science, Karolinska Institutet, Danderyd Hospital, Stockholm, Sweden; Karolinska Institutet, Department of Molecular Medicine and Surgery, Section of Orthopedics and Sports Medicine, Karolinska University Hospital, Stockholm, Sweden; Department of Orthopedics, Karolinska University Hospital, Stockholm, Sweden; Karolinska Institutet, Department of Clinical Science, Intervention and Technology (CLINTEC), Stockholm, Sweden

**Keywords:** Hip fracture, Nutritional supplementation, FFMI, Handgrip strength, HRQoL

## Abstract

**Background:**

The catabolic state that follows hip fracture contributes to loss of muscle mass and strength, that is sarcopenia, which impacts functional ability and health-related quality of life. Measures to prevent such long-term postoperative consequences are of important concern. The aim of this study was to evaluate the combined effects of protein-rich nutritional supplementation and bisphosphonate on body composition, handgrip strength and health-related quality of life following hip fracture.

**Methods:**

The study included 79 men and women with hip fracture, mean age 79 years (SD 9), without severe cognitive impairment, who were ambulatory and living independently before fracture. Patients were randomized postoperatively to receive liquid supplementation that provided 40 g of protein and 600 kcal daily for six months after the fracture, in addition to bisphosphonates once weekly for 12 months (group N, *n* = 26), or bisphosphonates alone once weekly for 12 months (group B, *n* = 28). All patients, including the controls (group C, *n* = 25) received calcium 1 g and vitamin D3 800 IU daily. Body composition as measured by dual-energy X-ray absorptiometry (DXA), handgrip strength (HGS) and health-related quality of life (HRQoL) were registered at baseline, six and 12 months postoperatively.

**Results:**

There were no differences among the groups regarding change in fat-free mass index (FFMI), HGS, or HRQoL during the study year. Intra-group analyses showed improvement of HGS between baseline and six months in the N group (*P* = 0.04). HRQoL decreased during the first year in the C and B groups (*P* = 0.03 and *P* = 0.01, respectively) but not in the nutritional supplementation N group (*P* = 0.22).

**Conclusions:**

Protein-rich nutritional supplementation was unable to preserve FFMI more effectively than vitamin D and calcium alone, or combined with bisphosphonate, in this relatively healthy group of hip fracture patients. However, trends toward positive effects on both HGS and HRQoL were observed following nutritional supplementation.

**Trial registration:**

Clinicaltrials.gov NCT01950169 (Date of registration 23 Sept 2013).

**Electronic supplementary material:**

The online version of this article (doi:10.1186/s12877-015-0144-7) contains supplementary material, which is available to authorized users.

## Background

Following hip fracture, as many as 25–30 % of patients have reportedly been unable to return to their previous living situation, while 28 % of those who were ambulatory before fracture are unable to walk 12 months postoperatively [1, 2]. This loss of function is probably due in part to muscle wasting and loss of muscle strength, i.e. sarcopenia [3]. Protein-energy malnutrition, which is common in elderly hip fracture patients [4, 5], most likely contributes to sarcopenia and loss of ambulatory capacity. The prevalence of low muscle mass among hip fracture patients has been shown to be between 20-85 %, depending on age and gender [6, 7].

Besides the age-related loss of muscle mass, the hip fracture trauma, presurgical fasting and hip fracture-associated immobilization cause adverse changes in body composition. As much as 5–6 % of muscle mass may be lost during the first year following fracture [8, 9]. This should be compared with the reported age-related loss of muscle mass, which seems to be fairly constant at a yearly rate of 1–2 % after age 50 [10]. Increased muscle wasting following hip fracture is likely to be one of several factors with a bearing on poor outcome like reduced function [1, 11]. The benefit of nutritional supplementation following hip fracture is not conclusive and sometimes the opposite is reported, as in the latest Cochrane Collaboration Review [12]. This review concluded that nutritional supplementation may potentially have beneficial effects, such as reducing general complications. However, only one randomized trial has previously evaluated the effects of protein and energy-rich supplementation on body composition as measured by DXA following hip fracture [13]. This study showed that muscle mass was protected from catabolism when protein supplementation was combined with an anabolic drug.

The hypothesis of the present study was that protein and energy-rich supplementation provided together with bisphosphonate may be able to limit muscle catabolism, have beneficial effects on muscle strength and thereby also improve health-related quality of life (HRQoL) following hip fracture in the elderly. Secondary specific objectives were to study the relationships between fat-free mass index (FFMI), appendicular lean mass index (aLMI) and handgrip strength (HGS).

## Methods

### Patients

The study included 79 patients, 56 women (71 %) and 23 men (29 %), mean age 79 (SD 9, range 61–96 years), who were admitted to one of the four university hospitals in Stockholm, Sweden, with the diagnosis of femoral neck or trochanteric fracture. As described previously, [14] inclusion criteria were age 60 or older, without severe cognitive impairment, ambulatory before fracture, living independently. From the beginning we included patients with BMI less than 26 kg/m^2^ since BMI of 26 is the average BMI in community dwelling men and women in Sweden aged > 70 years. Due to the difficulties in recruiting patients the inclusion cut-off was increased to 28 kg/m^2^.

Patients who had received bisphosphonate treatment in the year prior to admission, or who had pathological fracture, malignancy, or bone metabolic disorder were excluded. Further exclusion criteria were abuse of alcohol, drugs, or the presence of an obvious psychiatric disorder, dysphagia, esophagitis, gastric ulcer, lactose intolerance, diabetes mellitus with nephropathy or retinopathy, and active iritis or uveitis. Patients with abnormal liver and kidney function tests were also excluded.

### Study design and intervention

The primary purpose of this randomized multicenter trial was to study the effects of combined therapy with bisphosphonate and protein-rich nutritional supplementation after hip fracture on body composition, HGS and HRQoL. The outcome on bone mineral density of the same study population was recently reported [14]. To summarize the study design, patients were randomized into three treatment groups using sealed envelopes. Randomization was carried out by a research nurse, in collaboration with the trial physician in charge at each hospital. All patients received calcium 1 g and vitamin D 800 IE; specifically, cholecalciferol (Calcichew-D3®; Takeda Pharmaceutical Company Limited, Osaka, Japan) divided into two daily doses for 12 months. The nutritional supplementation group (protein + energy = N group) received a 200 ml package twice daily, each containing 20 g of protein and 300 kcal (Fresubin®, Fresenius Kabi, Bad Homburg, Germany). This supplement was given for the first six months following hip fracture and was combined with risedronate (Optinate® Septimum; Sanofi AB, Warner Chilcott, Weiterstadt, Germany), 35 mg once weekly for 12 months. The second group (B) received risedronate alone, 35 mg once weekly for 12 months. The controls (C) received only calcium and vitamin D for 12 months. Treatment began as soon as the patients were medically stable after surgery, able to take orally administered medications and able to sit upright for one hour after intake of bisphosphonates. Due to potential compliance problems, as well as anticipated effects on muscle mass and function within 6 months, it was decided to cease the nutritional supplementation after 6 months. Blood samples were collected in the morning of the first weekday on the ward. DXA and all calculations were carried out during the hospital stay and patients were re-examined at 6 and 12 months. All patients received conventional rehabilitation aimed at restoring the ability to walk.

Approval for the study was obtained from the local Ethics Committee at Karolinska University Hospital, Stockholm. The study was conducted in accordance with the Helsinki Declaration. Before inclusion all participants provided written consent to participate in the study.

### Measurements

Body composition, including lean mass composed of muscle, visceral organs and water (LM), fat mass (FM) and bone mineral content (BMC), was measured by whole body dual-energy X-ray absorptiometry (DXA) using either Hologic (Hologic, Inc Waltham, MA, USA) or GE Lunar (Madison, WI, USA) densitometers. The sum of LM and BMC represents fat-free mass (FFM). To normalize for body size, FFM was divided by height squared to calculate fat-free mass index (FFMI, kg/m^2^). Fat mass index (FMI, kg/m^2^) was calculated analogously [15]. Cut-offs for low FFMI were <17 kg/m^2^ for men and <15 kg/m^2^ for women [16]. Mathematically, the sum of FFMI and FMI equals BMI. Cut-offs for FFMI were based on currently available European reference values for body composition in a Swiss population, rounded to the nearest integer [16]. Thus, FFMI <10th percentile of the reference population was considered to be low [15, 16]. Patient height, weight and body mass index (BMI) were monitored. Height was measured in supine position. Weight was calculated from the sum of LM, FM and BMC obtained from the DXA measurements and defined as total body mass (kg). Data on lean mass from DXA of legs and arms were used to calculate appendicular skeletal muscle mass (kg). For the purposes of this assessment, the lean mass of the un-fractured leg multiplied by 2 was used at baseline to avoid overestimation in the fractured leg due to postoperative edema [6]. An appendicular lean mass index (aLMI, kg/m^2^) was calculated by dividing appendicular lean mass (aLM, kg) by height squared [17]. Cut-off points used for low aLMI were ≤7.23 kg/m^2^ for men and ≤5.67 kg/m^2^ for women [18].

Handgrip strength (HGS, kg) was measured in the dominant hand by hand dynamometer, (JAMAR 5030 J1; Sammons Preston, Rolyan, Bolingbrook, IL, USA) with the patient in a sitting position; the highest value of three was recorded. HGS measured by the Jamar dynamometer has been shown to have good reproducibility (r > 0.80) and reliability (r = 0.98) [19, 20]. Cut-off points used for low HGS strength were <30 kg in men and <20 kg in women [3].

Ocurrence of sarcopenia was defined according to the recent suggestion from the European Working Group on Sarcopenia in Older People (EWGSOP) [3], as a combination of low aLMI and reduced HGS in accordance with the above-mentioned cut-off values.

HRQoL was defined using the EQ-5D descriptive system and converted to a single summary index for each patient (EQ-5D_index)_ [21]. An EQ-5D_index_ of 0.00 indicated the worst possible state of health and a value of 1.00 the best. According to the Short Portable Mental Status Questionnaire (SPMSQ), only patients without severe cognitive impairment (≥3 correct answers on 10 questions) were included in the study [22]. General physical health was assessed by the attending anesthesiologist before surgery according to the American Society of Anesthesiologists (ASA) classification [23].

Biochemical measures considered relevant for this study were analyzed according to the standard hospital laboratory procedure in plasma (P) or serum (S) at baseline, 6 and 12 months, including blood hemoglobin (B-Hgb, g/L), c-reactive protein (S-CRP, mg/L), albumin (P-Alb, g/L), alanine aminotransferase (S-ALT, μkat/L), aspartate aminotransferase (S-AST, μkat/L), P-creatinine (μmol/L) and S-thyroid-stimulating hormone (S-TSH, mE/L). Glomerular filtration rate (GFR, ml/min) was estimated from P-Cystatin C (mg/L). As a nutritional biochemical marker, serum levels of insulin-like growth factor-I (S-IGF-I, μg/L) were analyzed by radioimmunoassay [24]. Because of the age dependence of S-IGF-I, the values were also expressed as SD scores calculated from the regression of IGF-I values in a healthy reference population. SD scores ± 2SD were considered to be within the age reference range [25, 26].

### Statistical methods

Statistical calculations were performed using SPSS version 22.0 for Windows (IBM, SPSS Statistics). Mean, standard deviation, median, range and percentage were used for descriptive purposes.

Univariate correlations at baseline between FFMI and aLMI, and between aLMI and HGS were analyzed using Pearson correlation coefficient and Spearman’s rank correlation coefficient, respectively. Non-parametric tests were used to analyze EQ-5D_index_ data. For biochemical measurement analyses, both parametric and non-parametric tests were used depending on distribution and type of variables. Chi-square test and Fisher’s exact test were used to compare the proportions of patients with sarcopenia in the treatment groups.

Statistical analyses for the outcome measures body composition, FFMI, FMI and HGS were carried out using covariance analysis (ANCOVA). The ANCOVA analyses included the exposure measures treatment groups and sex as fixed factors. Age and baseline values for FFMI, FMI and HGS were included as covariates. To have a sort of sensitivity analysis of the results, the complete cases population was analyzed and results for this population were presented. To have a complete and objective evaluation of data, the results of the analysis for the intention-to-treat (ITT) population were also presented. Missing data were processed for each of the outcome measures according to the hot-deck method [27]. This method replaces missing data with randomly assigned values taken from individuals that are stratified according to sex and age. ITT analyses were performed using the database with imputed data.

Calculations revealed that a sample size of 40 patients per group was required to detect a difference in lean mass between groups (power = 80 %, a = 0.05). However, the study was closed after 4 years due to difficulties in recruiting patients and the study was switched to an exploratory design.

## Results

**Fig. 1:**
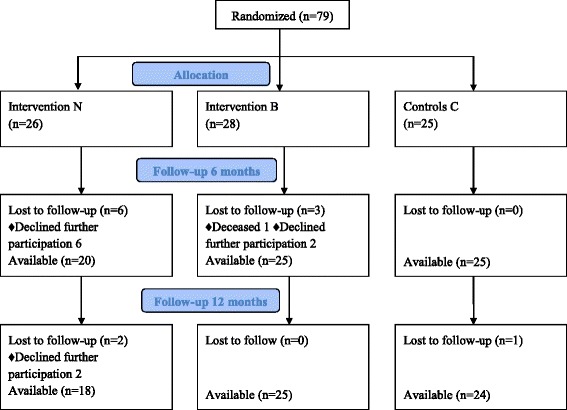
Flow chart of participants

The distribution of patients at inclusion and at follow-up is shown in the flow chart (Fig. 1). No significant difference in baseline characteristics was found between the groups (Table 1). Low FFMI was found in 22 of 79 (28 %) and low aLMI in 32 of 79 (40 %) patients at baseline. There was a positive correlation between FFMI and aLMI, r = 0.92, P <0.01. The total number of patients with sarcopenia, i.e. with both low aLMI and reduced HGS, was 16 of 75 (21 %) at baseline (missing HGS data *n* = 4); the corresponding figures at 6 and 12 months were 24 and 29 %, respectively, with no significant difference between groups over the observation period.

**Table 1:** Baseline characteristics of all participants and by intervention group

	All patients (*n* = 79)	Group N (*n* = 26)	Group B (*n* = 28)	Group C (*n* = 25)
Age, mean (SD)	79 (9)	81 (8)	80 (9)	78 (11)
Age, median (range)	81 (61–96)	82 (62–93)	82 (63–94)	75 (61–96)
Gender, female n (%)				
Women	56 (71)	19 (73)	18 (64)	19 (76)
Men	23 (29)	7 (27)	10 (36)	6 (24)
Type of fracture n (%)				
Femoral neck	33 (42)	10 (38)	12 (43)	11 (44)
Trochanteric	46 (58)	16 (62)	16 (57)	14 (56)
Surgical method n (%)				
Internal fixation	69 (87)	23 (88)	25 (89)	21 (84)
Arthroplasty	10 (13)	3 (12)	3 (11)	4 (16)
Time to surgery n (%)				
Within 24 hours	43 (54)	13 (16)	17 (22)	13 (16)
> 24 to 48 hours	36 (46)	13 (16)	11 (14)	12 (15)
ASA 1–2 n (%)	60 (76)	20 (25)	22 (28)	18 (23)
ASA 3–4 n (%)	19 (24)	6 (9)	6 (8)	7 (8)
Body mass,kg mean (SD)	63.0 (12.3)	61.4 (9.8)	66.9 (13.3)	60.2 (12.8)
Women	58.7 (10.0)	60.2 (9.8)	60.7 (10.0)	55.4 (9.8)
Men	73.3 (11.3)	64.7 (9.5)	78.2 (11.2)	75.4 (8.7)
BMI, kg/m^2^ mean (SD)	23.0 (3.0)	22.7 (3.4)	24.0 (2.9)	22.4 (2.6)
Women	22.8 (3.1)	23.0 (3.5)	23.9 (3.1)	21.7 (2.5)
Men	23.5 (2.8)	21.8 (3.3)	24.1 (2.8)	24.5 (1.2)
FFMI, kg/m^2^ mean (SD)	16.1 (2.0)	15.6 (1.9)	16.8 (2.0)	16.0 (2.0)
Women	15.3 (1.4)	15.0 (1.6)	15.9 (1.4)	15.1 (1.0)
Men	18.1 (2.0)	17.1 (2.0)	18.3 (2.0)	18.8 (1.7)
FMI, kg/m^2^ mean (SD)	6.9 (2.5)	7.1 (2.9)	7.2 (2.4)	6.4 (2.0)
Women	7.5 (2.5)	8.0 (2.6)	8.0 (2.4)	6.6 (2.2)
Men	5.5 (1.8)	4.8 (2.4)	5.8 (1.7)	5.7 (1.1)
aLMI, kg/m^2^	6.2 (0.9)	6.0 (0.8)	6.5 (0.9)	6.2 (1.0)
Women	5.9 (0.7)	5.8 (0.8)	6.1 (0.6)	5.8 (0.6)
Men	7.0 (1.0)	6.6 (80.8)	7.1 (1.0)	7.5 (0.9)
Handgrip strength, kg, median (range)	20 (6–64)	20 (6–63)	22 (6–64)	20 (8–58)
Women	18 (6–38)	18 (6–32)	18 (6–28)	17 (8–38)
Men	36 (20–64)	24 (20–33)	45 (28–64)	40 (23–58)
EQ-5D_index_ mean, (SD)	0.85 (0.21)	0.84 (0.22)	0.84 (0.25)	0.88 (0.12)
S-IGF-I, μg/L, median, (range)	87 (27–239)	72 (30–169)	93 (30–207)	87 (27–239)
S-IGF-I age matched SD- score	−0.6	−1.1	−0.1	−0.8
P-Alb g/L, median, (range)	34 (26–44)	34 (27–42)	34 (26–44)	34 (26–44)
B-Hbg g/L, median, (range)	124 (90–156)	124 (97–149)	128 (103–156)	119 (90–153)
S-CRP mg/L, median (range)	3 (0–257)	4 (0–136)	2 (0–253)	8 (0–257)
P-Creatinine μmol/L,median, (range)	71 (37–174)	69 (37–136)	74 (47–118)	71 (49–174)
S-ALAT, μkat/L, median, (range)	0.41 (0.10–8.82)	0.36 (0.10–8.82)	0.40 (0.12–3.02)	0.45 (0.19–1.57)
S-ASAT, μkat/L, median, (range)	0.65 (0.22–2.42)	0.66 (0.23–1.61)	0.62 (0.35–2.42)	0.60 (0.22–1.60)
S-TSH, mE/L, median (range)	1.5 (0.1–9.3)	1.7 (0.4–8.5)	1.4 (0.8–8.5)	1.4 (0.4–9.3)

Group N, received nutritional supplementation, bisphosphonate, calcium and vitamin D3; B, received bisphosphonate, calcium and vitamin D; C, controls, treated with calcium and vitamin D. Body mass (lean mass, fat mass and bone mineral content); ASA, the American Society of Anesthesiologists classification; BMI, body mass index; FFMI, fat free mass index; FMI, fat mass index; aLMI, appendicular lean mass index; EQ-5D_index_, the 5-Dimentional Scale of the HRQoL; S-IGF-I, serum-insulin growth factor-I; P-Alb, plasma-albumin; B-Hbg, blood-Hemoglobin; S-CRP, serum-C-reactive protein; P-creatinine, plasma creatinine; S-ALT, serum alanine aminotransferase; S-AST, serum aspartate aminotransferase; S-TSH, serum thyroid-stimulating hormone. Baseline characteristics showed no significant difference between the groups

### Treatment adherence with nutritional supplementation

As described previously [14], 11 of 18 patients presenting at the final follow-up reported consuming only half the prescribed intake of protein and energy drink (200 ml) giving an average of 0.32 g protein per kg bodyweight during the treatment period of 6 months. The remaining 7 patients did take the prescribed daily dose (200 ml *x*2) giving 0.70 g protein per kg bodyweight. There were no significant difference between those who were compliant to the prescription and those who took half prescribed supplement regarding outcome of total body mass, aLMI, FFMI, FMI or HGS at the two follow-ups (data not shown).

### Effect on body composition

An overall loss of body mass and FFM occurred during 6 and 12 months after fracture, with no significant difference between groups (Table 2). There were tendencies for increased FMI and a greater drop in FFMI and aLMI in the N group (Table 2). The ITT analyses confirmed this trend, showing no drop in FMI in the N group at 6 months (*P* = 0.01) and a greater drop of FFMI in the N group compared with the other two groups at 6 and 12 months (*P* <0.001 and *P* = 0.006, respectively).

**Table 2:** Outcome of body composition components, handgrip strength, health-related quality of life and biochemical measurements

	Months	All patients	Group N	Group B	Group C	*p*-value
Body mass, kg (SD)	^a^0-6	−2.1 (3.5)	−2.0 (3.5)	−3.0 (3.8)	−1.2 (3.2)	0.29
	^b^0-12	−1.6 (4.0)	−1.8 (3.0)	−2.2 (4.6)	−0.9 (4.1)	0.69
FFM, kg (SD)	^a^0-6	−1.5 (2.5)	−2.4 (2.0)	−1.3 (3.2)	−1.0 (1.9)	0.09
	^b^0-12	−1.6 (2.7)	−2.2 (2.5)	−1.4 (3.2)	−1.3 (2.2)	0.41
FM, Kg (SD)	^a^0-6	−0.6 (2.9)	+0.4 (2.3)	−1.7 (3.6)	−0.2 (2.1)	0.06
	^b^0-12	−0.2 (3.4)	+0.4 (2.1)	−0.7 (4.4)	−0.1 (3.0)	0.64
aLM, kg (SD)	^a^0-6	−0.1 (1.6)	−0.7 (1.4)	+0.3 (1.9)	+0.02 (1.4)	0.06
	^b^0-6	−0.2 (1.6)	−0.4 (1.5)	−0.02 (2.0)	−0.2 (1.4)	0.40
FFMI, kg/m^2^ (SD)	^a^0-6	−0.6 (0.9)	−0.9 (0.7)	−0.4 (1.2)	−0.4 (0.8)	0.08
	^b^0-12	−0.6 (1.0)	−0.8 (0.9)	−0.5 (1.2)	−0.5 (0.8)	0.31
FMI	^a^0-6	−0.2 (1.1)	0.1 (0.8)	−0.6 (1.4)	−0.1 (0.8)	0.06
kg/m^2^ (SD)	^b^0-12	−0.1 (1.2)	0.1 (0.8)	−0.3 (1.6)	−0.1 (1.1)	0.62
aLMI	^a^0-6	0.0 (0.6)	−0.2 (0.5)	+0.1 (0.7)	0.0 (0.6)	0.03
kg/m^2^ (SD)	^b^0-12	−0.1 (0.6)	−0.2 (0.5)	0.0 (0.7)	−0.1 (0.5)	0.30
HGS, kg (SD)	^c^0-6	1.4 (4.8)	2.6 (4.7)	−0.3 (4.0)	2.2 (5.4)	0.09
	^d^0-12	−1.0 (5.8)	−0.06 (6.6)	−3.0 (6.1)	−0.2 (5.7)	0.22
EQ-5D_index_ (SD)	^c^6	0.77 (0.23)	0.75 (0.32)	0.76 (0.20)	0.78 (0.19)	0.57
	^d^12	0.74 (0.23)	0.76 (0.22)	0.70 (0.25)	0.75 (0.24)	0.60
S-IGF-I,μg/L (range)	6	124 (31–280)	124 (39–265)	135 (45–280)	117 (31–236)	0.39
	12	119 (45–310)	111 (45–205)	137 (68–310)	129 (66–202)	0.35
S-IGF-I, SD score	6	0.4	0.4	0.6	0.1	0.42
	12	0.2	0.1	0.7	0.4	0.58
P-Alb, g/L (range)	6	38 (24–47)	37 (28–46)	38 (24–42)	39 (33–47)	0.48
	12	38 (28–46)	40 (29–46)	37 (34–45)	39 (33–45)	0.31
B-Hgb, g/L (range)	6	134 (107–171)	136 (110–171)	134 (113–160)	132 (107–154)	0.89
	12	136 (109–162)	136 (109–162)	134 (115–153)	136 (115–151)	0.84
S-CRP, mg/L (range)	6	0 (0–48)	4 (0–32)	0 (0–48)	0 (0–17)	0.15
	12	1 (0–21)	2 (0–21)	1 (0–8)	1 (0–6)	0.24
P-Creatinine, μmol/L (range)	6	71 (41–173)	67 (41–123)	76 (55–117)	72 (50–173)	0.38
	12	73 (41–173)	67 (43–127)	80 (57–128)	71 (49–118)	0.19

Group N = protein and energy supplementation for 6 months, and risedronate, vitamin D and calcium for 12 months, B = risedronate, vitamin D and calcium for 12 months, C = vitamin D and calcium for 12 months. Body mass (lean mass, fat mass and bone mineral content); FFM, fat free mass; FM, fat mass; aLM, appendicular lean mass; FFMI, fat free mass index; FMI, fat mass index; HGS, handgrip strength; EQ-5D_index_, the 5-Dimentional Scale of the HRQoL; S-IGF-I, serum-insulin growth factor-I; P-Alb, plasma-Albumin; B-Hgb, blood-Hemoglobin; S-CRP, serum-C-reactive protein; P-Creatinine, plasma-Creatinine. Data are given as mean (SD) or median (range). ^a^Patients analyzed 0–6 months in group N, *n* = 19; group B, *n* = 25; group C, *n* = 24. ^b^Patients analyzed 0–12 months in group N, *n* = 18; group B, *n* = 25; Group C; *n* = 23. ^c^Patients analyzed 0–6 months in group N, *n* = 20; group B, *n* = 25; group C, *n* = 25. ^d^Patients analyzed 0–12 months in group N, *n* = 18; group B, *n* = 25; group C, *n* = 21

### Effect on handgrip strength

**Fig. 2:**
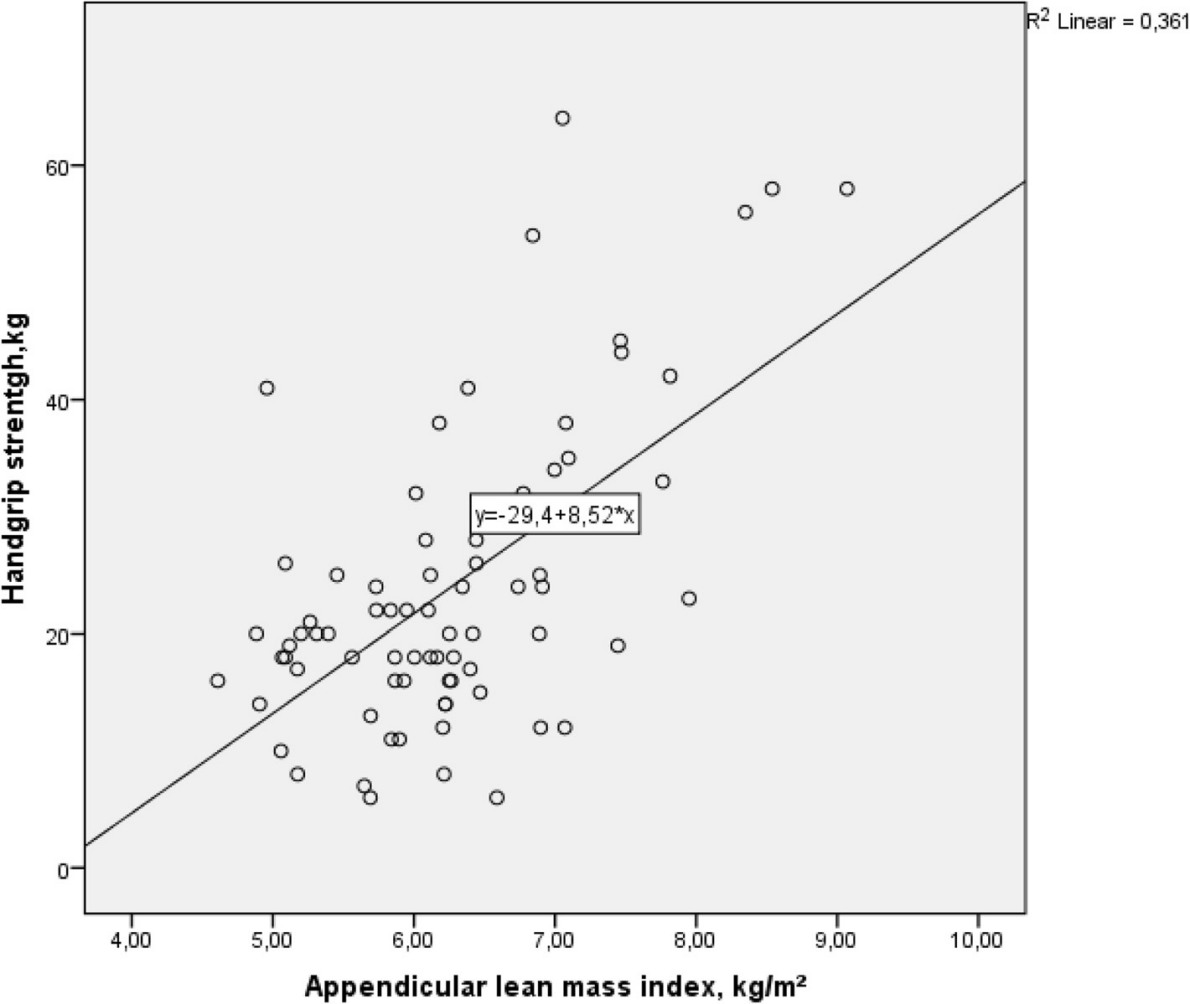
Relationship between appendicular lean mass index and handgrip strength at baseline

A positive correlation was found between aLMI and HGS at baseline (r_s_ = 0.47, *P* < 0.01, Fig. 2), and at 6 (r_s_ = 0.61, *P* < 0.01) and 12 months (r_s_ = 0.64, *P* < 0.01). A trend for improved HGS was seen (complete cases, table 2), but was not confirmed by ITT analysis. Intra-group analysis showed a significant increase in HGS within the N group during the first 6 months (*P* = 0.04), while this change was not significant for the other two groups.

### Effect on HRQoL

**Fig. 3:**
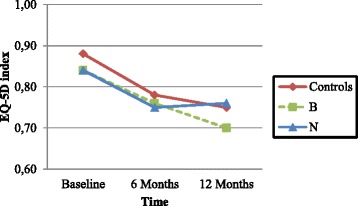
Outcome of EQ-5D index in different treatment groups at baseline, 6 and 12 months postoperatively. Group C, Controls = vitamin D and calcium for 12 months; B = risedronate, vitamin D and calcium for 12 months; *N* = protein and energy supplementation for 6 months, and risedronate, vitamin D and calcium for 12 months

The EQ-5D_index_ decreased from 0.85 (SD 0.21) at baseline to 0.77 (SD 0.23) at 6 months and 0.74 (SD 0.23) at 12 months for all patients. Inter-group analysis showed no differences (Table 2). Intra-group analysis between 0–12 months showed a significant decrease in the EQ-5D_index_ for the C and B groups (*P* = 0.03 and *P* = 0.01, respectively), but not for the N group (*P* = 0.22) (Fig. 3).

### Biochemical measures

The biochemical measures showed no significant differences between groups at baseline (Table 1), or at the two follow-ups (Table 2). Average S-IGF-I and age-adjusted SD score were within normal range in all groups at baseline (Table 1). S-IGF-I increased between baseline and 6 months in all groups without inter-group differences (Table 2).

## Discussion

This study evaluated the potential for protein and energy supplementation combined with bisphosphonate to preserve muscle mass when prescribed to elderly hip fracture patients over an extended period of time. However, this intervention, which was administered together with vitamin D and calcium, had no positive effect on handgrip strength, HRQoL, or lean mass, when compared with administration of bisphosphonate along with vitamin D and calcium supplementation, or just vitamin D and calcium supplementation alone after hip fracture. Thus, none of the study hypotheses could be confirmed. The results of the intention-to-treat analysis were in line with those of the complete-cases analysis. However, intra-group analysis did show a positive effect on HRQoL and improved HGS in the nutritional supplementation group. These intra-group differences may indicate that the study was underpowered to answer the questions addressed. Post-hoc analyses showed that at least 135 patients (vs the 79 in this study) would be needed to reach statistical significance between groups for effect on HGS. Furthermore, because emerging evidence suggests that vitamin D has positive effects on muscle strength, administration of vitamin D to all groups may have negated any significant differences in treatment effects among groups [28].

The finding of postoperative catabolism with loss of lean mass despite protein-rich nutritional supplementation following hip fracture is consistent with a previous study [13]. Loss of lean mass in the prior study was 1.6 kg (SD 1.5) and in the present 2.1 kg (SD 2.6) in the first year after fracture, despite nutritional supplementation in both studies. The prior study showed that lean mass was preserved during the first 6 months only among patients who received an anabolic steroid in addition to supplementation [13]. Loss of lean mass following hip fracture has been reported at 5–6 % during the first year [8, 9], which is in line with the observed loss of 5.2 % in our study, even though our patients received nutritional support for 6 months. It could be debated if the nutritional supplementation should have been lasting longer than six months, however the idea was to attenuate lean mass loss during the first six postoperative months when the effects of trauma and surgery on weight and lean mass is most pronounced. Considering compliance, six months was also a reasonable time for an intervention study with nutritional supplementation. As far as we know there has not been any nutritional intervention trials earlier persisting longer than six months for hip fracture patients. One explanation for the lack of significant benefits may be that the prolonged catabolic state, with its metabolic, hormonal and inflammatory response to trauma and surgery, results in an accelerated and prolonged breakdown of muscle protein [29, 30]. This negative energy balance in response to injury may also have a negative impact on the ability of patients to benefit from nutrients [29, 31]. Many hip fracture patients suffer from poor nutrition and a negative energy balance prior to admission for hip fracture [5]. One previous study showed no improvement in nutritional status, despite adequate energy intake postoperatively among patients who were undernourished prior to hip fracture surgery [32]. Recently, there has been increased focus on the composition of protein supplementation. Indications have emerged that certain essential amino acids, specifically the branched-chain amino acid leucine, which is abundant in whey protein or its metabolite hydroxy-methyl-butyrate (HMB), may have anabolic effects in addition to the purely nutritional effects [33, 34]. This study used a traditional balanced mix of proteins. Further studies are needed to assess whether more explicit beneficial effects could be attained by use of these specific protein compounds.

The proportion of patients with sarcopenia did not differ statistically among the groups over the observation period. The prevalence of sarcopenia in total was 21 % at baseline, and 24 and 29 %, respectively after 6 and 12 months. This confirms earlier reports of a catabolic situation that persists during the first year after hip fracture [35]. The proportion of hip fracture patients with low aLMI at baseline has previously been reported to be 47 %, compared with 40 % in the present study [7]. This slight difference may be due to the selection of a relatively healthy group of patients in the present study and also to the use of different normative data. We used the aLMI reference values from a study by Newman et al. [18], while the previous study used normative aLMI data derived from a Japanese population [36]. We found a higher proportion of patients with low aLMI at both 6 and 12 months than at baseline, but no previous studies with aLMI results at one year following hip fracture are available for comparison with our findings.

A trend toward preserved FMI was seen at 6 months in the nutritional supplementation group, which was significantly supported by the intention-to-treat analysis, and was also seen in a previous study with nutritional support prior to elective hip surgery [37]. A possible explanation for preserved FMI, albeit not FFMI, through nutritional supplementation in the present study might be the impact that lack of resistance training during rehabilitation could have on fat metabolism. Exercise programs with resistance training combined with nutritional supplementation have been reported to result in lean mass gain [38]. Still an increase in FMI may indicate some anabolic effects of the supplementation. The interactions between fat mass, fat free mass and function need to be better understood.

We noted improved HGS in the protein and energy supplementation group between baseline and 6 months. A similar improvement in HGS after 3 months of nutritional supplementation has been shown earlier in elderly patients following hospitalization due to acute illness, as well as in chronically-ill outpatients [39, 40]. Strength or physical performance is a complex parameter that relates to more than just mass, meaning that muscle function may improve even when muscle mass remains unaffected [41, 42]. This may help explain our findings in the nutritional supplementation group showing a trend toward preserved HGS, but not FFMI, as well as the modest relationship between HGS and aLMI found in our analysis. We chose to measure HGS in the present study since a strong association has been shown between HGS and muscle strength in the legs, and also because HGS actually predicts mobility better than muscle mass [43].

HRQoL decreased in all groups and failed to reach pre-fracture levels by 12 months, consistent with an earlier study following hip fracture [44]; however, intra-group analysis showed less of a drop in the nutritional supplementation group. Preservation of HRQoL after hip fracture has not previously been seen following supplementation alone, but has been demonstrated when supplementation is combined with an anabolic steroid [13].

Certain limitations in this study need to be acknowledged. As mentioned above, one major limitation is the small number of study subjects, which may lead to type 2 errors, i.e. the risk of missing a true positive effect. The difficulties of including large numbers of hip fracture patients in intervention studies with nutritional supplementation are generally acknowledged [12]. However, in our opinion this study might make a substantial contribution if meta-analysis is undertaken in the future. Other limitations include the high attrition rate and suboptimal adherence in the nutritional supplementation group, both of which have been encountered in earlier studies [45, 46]. The use of different devices of DXA measurements inflict uncertainties on the validity of the results. Still, this is a problem multi-center studies are usually facing and difficult to avoid. Further, intervention was first initiated postoperatively, which may have reduced the ability to counter the effects of the catabolic process that was already underway before surgery. The strength and novelty of this study lie in the DXA measurements of body composition with assessment of changes in fat-free mass, fat mass, appendicular muscle mass, strength and HRQoL, to evaluate the response to oral nutritional supplementation following hip fracture in the elderly. Other attributes include assessment of appendicular lean mass index together with HGS to determine the prevalence of sarcopenia in the study population not just at baseline, but also postoperatively for one year. The randomized design and the long 12-month follow-up period were additional strengths.

## Conclusions

In this fairly small study, supplementation with protein and energy combined with conventional rehabilitation were not able to preserve lean mass following hip fracture any better than vitamin D and calcium alone, or combined with bisphosphonates. There were no inter-group differences concerning effects on HGS or HRQoL, but intra-group improvement in HGS and a positive effect on HRQoL were seen in the nutritional supplementation group.

## Additional file

Additional file 1:
**CONSORT checklist for RCT protocols.** (DOC 38 kb)

## Abbreviations

aLM: appendicular Lean Mass
aLMI: appendicular Lean Mass Index
ASA: The American Society of Anesthesiologists Classification
BMC: Bone Mineral Content
BMI: Body Mass Index
DXA: Dual-energy X-ray Absorptiometry
EQ-5D: The 5-Dimentional Scale of the HRQoL
EWGSOP: European Working Group on Sarcopenia in older People
FFM: Fat-Free Mass
FFMI: Fat-Free Mass Index
FM: Fat Mass
FMI: Fat Mass Index
HGS: Handgrip Strength
HRQoL: Health-Related Quality of Life
ITT: Intention-To-Treat
LM: Lean Mass
SPMSQ: Short Portable Mental Status Questionnaire

## References

[1] Al-Ani AN, Flodin L, Soderqvist A, Ackermann P, Samnegard E, Dalen N, et al. Does rehabilitation matter in patients with femoral neck fracture and cognitive impairment? A prospective study of 246 patients. Arch Phys Med Rehabil. 2010;91(1):51–7. 10.1016/j.apmr.2009.09.005 20103396

[2] Samuelsson B, Hedstrom MI, Ponzer S, Soderqvist A, Samnegard E, Thorngren KG, et al. Gender differences and cognitive aspects on functional outcome after hip fracture--a 2 years’ follow-up of 2,134 patients. Age Ageing. 2009;38(6):686–92. 10.1093/ageing/afp169 19767316

[3] Cruz-Jentoft AJ, Baeyens JP, Bauer JM, Boirie Y, Cederholm T, Landi F, et al. Sarcopenia: European consensus on definition and diagnosis: Report of the European Working Group on Sarcopenia in Older People. Age Ageing. 2010;39(4):412–23. 10.1093/ageing/afq034 PMC2886201 20392703

[4] Bachrach-Lindstrom MA, Ek AC, Unosson M (2000). Nutritional state and functional capacity among elderly Swedish people with acute hip fracture. Scand J Caring Sci.

[5] Bell J, Bauer J, Capra S, Pulle CR (2013). Barriers to nutritional intake in patients with acute hip fracture: time to treat malnutrition as a disease and food as a medicine?. Can J Physiol Pharmacol.

[6] Di Monaco M, Castiglioni C, Vallero F, Di Monaco R, Tappero R (2012). Sarcopenia is more prevalent in men than in women after hip fracture: a cross-sectional study of 591 inpatients. Arch Gerontol Geriat.

[7] Hida T, Ishiguro N, Shimokata H, Sakai Y, Matsui Y, Takemura M, et al. High prevalence of sarcopenia and reduced leg muscle mass in Japanese patients immediately after a hip fracture. Geriatr Gerontol Int. 2013;13(2):413–20. 10.1111/j.1447-0594.2012.00918.x 22816427

[8] Fox KM, Magaziner J, Hawkes WG, Yu-Yahiro J, Hebel JR, Zimmerman SI, et al. Loss of Bone Density and Lean Body Mass after Hip Fracture. Osteoporosis Int. 2000;11(1):31–5. 10.1007/s001980050003 10663356

[9] Karlsson M, Nilsson JA, Sernbo I, Redlund-Johnell I, Johnell O, Obrant KJ, et al. Changes of bone mineral mass and soft tissue composition after hip fracture. Bone. 1996;18(1):19–22. 10.1016/8756-3282(95)00422-x 8717532

[10] Hughes VA, Frontera WR, Roubenoff R, Evans WJ, Singh MA (2002). Longitudinal changes in body composition in older men and women: role of body weight change and physical activity. Am J Clin Nutr.

[11] Koval KJ, Skovron ML, Aharonoff GB, Meadows SE, Zuckerman JD (1995). Ambulatory ability after hip fracture. A prospective study in geriatric patients. Clin Orthop Rel Res.

[12] Avenell A, Handoll HH (2010). Nutritional supplementation for hip fracture aftercare in older people. Cochrane Database Syst Rev (Online).

[13] Tidermark J, Ponzer S, Carlsson P, Soderqvist A, Brismar K, Tengstrand B, et al. Effects of protein-rich supplementation and nandrolone in lean elderly women with femoral neck fractures. Clin Nutr. 2004;23(4):587–96. 10.1016/j.clnu.2003.10.006 15297095

[14] Flodin L, Saaf M, Cederholm T, Al-Ani AN, Ackermann PW, Samnegard E, et al. Additive effects of nutritional supplementation, together with bisphosphonates, on bone mineral density after hip fracture: a 12-month randomized controlled study. Clin Interv Aging. 2014;9:1043–50. 10.2147/CIA.S63987 PMC4094579 25045257

[15] Schutz Y, Kyle UU, Pichard C (2002). Fat-free mass index and fat mass index percentiles in Caucasians aged 18–98 y. Int J Obesity.

[16] Kyle UG, Schutz Y, Dupertuis YM, Pichard C (2003). Body composition interpretation. Contributions of the fat-free mass index and the body fat mass index. Nutrition.

[17] Baumgartner RN, Koehler KM, Gallagher D, Romero L, Heymsfield SB, Ross RR, et al. Epidemiology of sarcopenia among the elderly in New Mexico. Am J Epidemiol. 1998;147(8):755–63. 10.1093/oxfordjournals.aje.a009520 9554417

[18] Newman AB, Kupelian V, Visser M, Simonsick E, Goodpaster B, Nevitt M, et al. Sarcopenia: alternative definitions and associations with lower extremity function. J Am Geriatr Soc. 2003;51(11):1602–9. 10.1046/j.1532-5415.2003.51534.x 14687390

[19] Bohannon RW, Schaubert KL (2005). Test-retest reliability of grip-strength measures obtained over a 12-week interval from community-dwelling elders. J Hand Ther.

[20] Peolsson A, Hedlund R, Oberg B (2001). Intra- and inter-tester reliability and reference values for hand strength. J Rehabil Med.

[21] Burstrom K, Johannesson M, Diderichsen F (2001). Swedish population health-related quality of life results using the EQ-5D. Qual Life Res.

[22] Pfeiffer E (1975). A short portable mental status questionnaire for the assessment of organic brain deficit in elderly patients. J Am Geriatr Soc.

[23] Owens WD, Felts JA, Spitznagel EL (1978). ASA physical status classifications: a study of consistency of ratings. Anesthesiol.

[24] Bang P, Eriksson U, Sara V, Wivall IL, Hall K (1991). Comparison of acid ethanol extraction and acid gel filtration prior to IGF-I and IGF-II radioimmunoassays: improvement of determinations in acid ethanol extracts by the use of truncated IGF-I as radioligand. Acta Endocrinol.

[25] Hilding A, Hall K, Wivall-Helleryd IL, Saaf M, Melin AL, Thoren M (1999). Serum levels of insulin-like growth factor I in 152 patients with growth hormone deficiency, aged 19–82 years, in relation to those in healthy subjects. J Clin Endocrinol Metab.

[26] Unden AL, Elofsson S, Knox S, Lewitt MS, Brismar K (2002). IGF-I in a normal population: relation to psychosocial factors. Clin Endocrinol.

[27] Andridge RR, Little RJ (2010). A review of hot deck imputation for survey non-response. Int Statist Rev.

[28] Muir SW, Montero-Odasso M (2011). Effect of vitamin D supplementation on muscle strength, gait and balance in older adults: a systematic review and meta-analysis. J Am Geriatr Soc.

[29] Ljungqvist O, Soop M, Hedstrom M (2007). Why metabolism matters in elective orthopedic surgery: a review. Acta Orthop.

[30] Patterson BM, Cornell CN, Carbone B, Levine B, Chapman D (1992). Protein depletion and metabolic stress in elderly patients who have a fracture of the hip. J Bone Joint Surg American volume.

[31] Hebuterne X, Bermon S, Schneider SM (2001). Ageing and muscle: the effects of malnutrition, re-nutrition, and physical exercise. Curr Opin Clin Nutr Metab Care.

[32] Paillaud E, Bories PN, Le Parco JC, Campillo B (2000). Nutritional status and energy expenditure in elderly patients with recent hip fracture during a 2-month follow-up. Brit J Nutr.

[33] Kim HK, Suzuki T, Saito K, Yoshida H, Kobayashi H, Kato H, et al. Effects of exercise and amino acid supplementation on body composition and physical function in community-dwelling elderly Japanese sarcopenic women: a randomized controlled trial. J Am Geriatr Soc. 2012;60(1):16–23. 10.1111/j.1532-5415.2011.03776.x 22142410

[34] Stout JR, Smith-Ryan AE, Fukuda DH, Kendall KL, Moon JR, Hoffman JR, et al. Effect of calcium beta-hydroxy-beta-methylbutyrate (CaHMB) with and without resistance training in men and women 65 + yrs: a randomized, double-blind pilot trial. Exp Gerontol. 2013;48(11):1303–10. 10.1016/j.exger.2013.08.007 23981904

[35] Hedstrom M. Hip fracture patients, a group of frail elderly people with low bone mineral density, muscle mass and IGF-I levels. Acta Physiol. 1999;167(4):347–7. 10.1046/j.1365-201x.1999.00626.x 10632638

[36] Sanada K, Miyachi M, Tanimoto M, Yamamoto K, Murakami H, Okumura S, et al. A cross-sectional study of sarcopenia in Japanese men and women: reference values and association with cardiovascular risk factors. Eur J Appl Physiol. 2010;110(1):57–65. 10.1007/s00421-010-1473-z 20390291

[37] Aronsson A, Al-Ani NA, Brismar K, Hedstrom M (2009). A carbohydrate-rich drink shortly before surgery affected IGF-I bioavailability after a total hip replacement. A double-blind placebo controlled study on 29 patients. Aging Clin Exp Res.

[38] Tieland M, Dirks ML, van der Zwaluw N, Verdijk LB, van de Rest O, de Groot LC, et al. Protein supplementation increases muscle mass gain during prolonged resistance-type exercise training in frail elderly people: a randomized, double-blind, placebo-controlled trial. J Am Med Dir Assoc. 2012;13(8):713–9. 10.1016/j.jamda.2012.05.020 22770932

[39] Cederholm TE, Hellstrom KH (1995). Reversibility of protein-energy malnutrition in a group of chronically-ill elderly outpatients. Clin Nutr.

[40] Price R, Daly F, Pennington CR, McMurdo ME (2005). Nutritional supplementation of very old people at hospital discharge increases muscle strength: a randomised controlled trial. Gerontology.

[41] Goodpaster BH, Park SW, Harris TB, Kritchevsky SB, Nevitt M, Schwartz AV, et al. The loss of skeletal muscle strength, mass, and quality in older adults: the health, aging and body composition study. J Gerontol A Biol Sci Med Sci. 2006;61(10):1059–64. 10.1093/gerona/61.10.1059 17077199

[42] Tieland M, van de Rest O, Dirks ML, van der Zwaluw N, Mensink M, van Loon LJ, et al. Protein supplementation improves physical performance in frail elderly people: a randomized, double-blind, placebo-controlled trial. J Am Med Dir Assoc. 2012;13(8):720–6. 10.1016/j.jamda.2012.07.005 22889730

[43] Lauretani F, Russo CR, Bandinelli S, Bartali B, Cavazzini C, Di Iorio A, et al. Age-associated changes in skeletal muscles and their effect on mobility: an operational diagnosis of sarcopenia. J Appl Physiol. 2003;95(5):1851–60. 10.1152/japplphysiol.00246.2003 14555665

[44] Ekstrom W, Miedel R, Ponzer S, Hedstrom M, Samnegard E, Tidermark J (2009). Quality of life after a stable trochanteric fracture--a prospective cohort study on 148 patients. J Orthop Trauma.

[45] Bruce D, Laurance I, McGuiness M, Ridley M, Goldswain P (2003). Nutritional supplements after hip fracture: poor compliance limits effectiveness. Clin Nutr.

[46] Miller MD, Daniels LA, Bannerman E, Crotty M (2005). Adherence to nutrition supplements among patients with a fall-related lower limb fracture. Nutr Clin Pract.

